# A Sandwich-Type Electrochemical Immunosensor Using Antibody-Conjugated Pt-Doped CdTe QDs as Enzyme-Free Labels for Sensitive HER2 Detection Based on a Magnetic Framework

**DOI:** 10.3389/fchem.2022.881960

**Published:** 2022-06-09

**Authors:** Hosna Ehzari, Meysam Safari

**Affiliations:** Department of Chemical Engineering, Kermanshah University of Technology, Kermanshah, Iran

**Keywords:** human epidermal growth factor receptor 2, sandwich-type electrochemical immunosensor, metal–organic frameworks, quantum dots, magnetic framework

## Abstract

Tumor markers are highly sensitive and play an important role in the early diagnosis of cancer. We developed an electrochemical sandwich-type immunosensor that detects human epidermal growth factor receptor 2 (HER2). Magnetic framework (Fe_3_O_4_@ TMU-24) and AuNPs (Fe_3_O_4_@ TMU-24 -AuNPs) are utilized in this sensing platform. In addition to their high specific surface area and excellent biocompatibility, Fe_3_O_4_@ TMU-24-AuNPs nanocomposites exhibited excellent electrocatalytic properties. The primary antibody of HER2 (Ab_1_) was immobilized on the surface of the Fe_3_O_4_@ TMU-24-AuNPs. In this sensing method, palatine doped to CdTe QDs (Pt: CdTe QDs) is utilized as a novel labeling signal biomolecule (secondary antibodies). Pt: CdTe QDs own good biocompatibility and excellent catalytic performance. The amperometric technique was used to achieve the quantitative determination of HER2 by using a sandwich-type electrochemical immunosensor. Under the optimum conditions, the dependency of the current signal and HER2 concentration showed a linear region from 1 pg ml^−1^–100 ng ml^−1^ with 0.175 pg ml^−1^ as the limit of detection. This biosensing device also showed long stability and good reproducibility, which can be used for the quantitative assay of HER2.

## Introduction

Human epidermal growth factor receptor 2 (HER2) is a member of the epidermal growth factor receptor family that facilitates the overgrowth or uncontrolled growth of breast cancer cells. HER2 overexpression of cancers of the breast, ovary, bladder, pancreas, and stomach occurs and acts as a prognostic and predictive biomarker (Ehzari et al., 2020c). HER2 has also been found in other cancers such as ovarian, endometrial, bladder, lung, colon, and head and neck (Slamon et al., 2018). In about one in five breast cancers, the cancer cells have extra copies of the gene that makes the HER2 protein. On the other hand, HER2-positive cancers grow and spread faster than other types of cancers. Therefore, the introduction of HER2-guided therapies can have a positive effect on the treatment of patients with breast and stomach/stomach cancer with HER2 (Scaltriti et al., 2007; Campone et al., 2019). There are currently several laboratory techniques used for the detection of HER2. For example, immunofluorescence, fluorescent *in situ* hybridization (FISH) (Press et al., 2019), enzyme-linked immunosorbent assay (ELISA), and immunohistochemistry (IHC) (Agersborg et al., 2018) are time consuming, costly, and difficult to perform.

Hence, an early and inexpensive diagnosis of cancer, which is critical for clinical diagnosis and immediate monitoring, can significantly reduce mortality. Electrochemical immunosensor with unique features in terms of inherent high sensitivity, simplicity, downsizing, real-time monitoring, etc. attracted a lot of attention and is widely used in the electrochemical detection of biomarkers (Wang et al., 2019). It is particularly the sandwich-type electrochemical immunosensor based on high specificity between antigen and antibody that is widely used in both clinical diagnosis and biochemical analysis due to its advantageous features involving rapid response, low detection limits, high sensitivity, ease of operation, and low manufacturing cost (Ehzari et al., 2020a).

It is a challenge to develop new approaches that can improve the sensitivity of clinical immunoassays, stability, and simplicity. The use of nanomaterials in biosensors has made them more stable, selective, and sensitive, and they reduce the cost of measurement because they increase the sensitivity and selectivity of biosensors (Prasad et al., 2019; Ehzari et al., 2021). Recently, electrochemical immunosensor using nanocrystals have been developed to detect biomarkers more rapidly and with greater sensitivity (Zhang et al., 2019; Ehzari et al., 2020a).

Metal–organic frameworks (MOFs) are a new generation of nanoporous coordinate polymers composed of metal clusters attached to organic bonds. The structure and physical and chemical properties of these materials depend on the chemical structure of the ligands and bonding metals (Jiang et al., 2021). Compared to other porous materials such as silica, activated carbon, and zeolites, MOFs have a flexible structure and a high surface area, which led to significant development in many fields including clean energy storage, hydrogen, environmental applications, sensors, removal and separation of toxic substances, medical and biological applications, catalysts, and solar cells (Safari et al., 2018; Yamini et al., 2018; Yamini and Safari, 2018, 2019; Hazrati and Safari, 2020; Karamipour et al., 2021; Safari and Yamini, 2021). The entry of metal ions into the structure of MOFs as a structural unit in coordination polymers determines the topology of the framework. On the other hand, due to the electron properties, the redox capabilities of d orbital electrons in these polymers have improved the performance of electrochemical and fluorescence sensors. The most commonly reported intermediates in coordination polymers are those that exhibit active metal–ligand bonds. These metals include manganese, cobalt, iron, copper, cadmium, nickel, silver, gold, zinc, and mercury. Depending on the metal element and its capacity, several geometric structures are possible (Zhang et al., 2018; Qiu et al., 2019; Ehzari et al., 2020b).

Thus, we designed an ultrasensitive electrochemical immunosensor AuNPs as a sensing platform and Pt: CdTe QDs as signal labels. Fe_3_O_4_@ TMU-24 various grafting groups (e.g., -NH_2_ or -COOH), π-π stacking, hydrogen bonding, and electrostatic interactions with an antibody have become a strong platform for the immunosensor. On the other hand, stand out with excellent stability, non-toxic property, and redox activity with Fe in their crystalline structure that can accelerate the movement of electrons, which ultimately improves the electrochemical signal and sensitivity toward the determination of HER2. An immunosensor based on the proposed design demonstrated excellent linear range, low detection limit, excellent specificity, reliable reproducibility, and acceptable stability.

## Experimental

### Reagents and Chemicals

Human serum albumin (HSA), HER2 antigen (human-HER2 mouse monoclonal), and HER2 antibody (anti-HER2-rabbit IgG) were purchased from Sigma (Sigma-Aldrich, United States). Bovine Serum Albumin (BSA), K_4_Fe(CN)_6_, K_3_Fe(CN)_6_, mercaptoacetic acid (MAA), mercaptoethanol, and aminobenzoic acid were obtained from Sigma-Aldrich. HAuCl_4_ 4H_2_O, methacrylic acid, 1,5-diaminonaphthalene, ammonium hydroxide (25 wt%), 4-pyridine carboxaldehyde, *ferrous chloride tetrahydrate*, and 4,4′-oxybisbenzoic acid (H_2_oba) were purchased from Merck (Darmstadt, Germany).

### Apparatus and Measurements

Electrochemical studies were carried out at room temperature using an Autolab potentiostat–galvanostat model PGSTAT30 using the NOVA software (version 1.11). A conventional three-electrode system composed of the modified GCE/Fe_3_O_4_@ TMU-24-AuNPs/MCA/Ab_1_ as the working electrode, a platinum wire as the counter electrode, and Ag/AgCl (saturated KCl) electrode as the reference electrode was used for all measurements. The Fourier transform infrared (FT-IR) was investigated in the transmission mode by Thermo Scientific Nicolet IR100 (Madison, WI, United States). A scanning electron microscope (SEM) (Philips instrument, Model XL 30) and energy-dispersive X-ray spectroscopy (EDX) was used to evaluate the modified surface electrode morphology.

### Preparation of Magnetic Framework Fe_3_O_4_@ TMU-24

Step-by-step assembly strategies were used to construct the magnetic framework composites (MFCs). Under stirring for 24 h, 0.3 g of Fe_3_O_4_ was added to 50 ml of ethanol solution containing 0.29 mM of MAA. The obtained Fe_3_O_4_@MAA was collected by magnetic separation and washed five times with DMF. Products were dispersed in 10 ml of 45 mM Zn(NO_3_)_2_.6H_2_O DMF solution for 30 min, followed by dispersion in 10 ml of a 4-nbpy and H_2_oba DMF solution (45 mM) for 30 min. Magnets were used to collect the resulting products between each step. Both of these steps were repeated 15 times. Fe_3_O_4_@TMU-24 is finally washed and dried under vacuum at 80°C.

### Synthesis of Pt: CdTe

The general approach for the synthesis of Pt: CdTe d-dots was performed by the previous procedure (Najafi et al., 2018; Safari et al., 2019). At first, sodium hydrogen telluride (NaHTe) was prepared by reducing Te powder with NaBH_4_ in deionized water under stirring conditions and N_2_ purging. After 3 h, the freshly NaHTe nanoparticle was prepared. In another flask, 0.3 g of CdCl_2_.5H_2_O and 0.2 mg of Na_2_PtCl_4_ were dissolved in 40 ml ultrapure water and after the addition of 200 µl of TGA under stirring conditions, its pH was adjusted to 10 by adding dropwise of NaOH solution (1 M) under N_2_ purging. Both free oxygen solutions were mixed and transferred into a Teflon-lined stainless steel autoclave and heated in an oven at 120°C for 3 h. At the end of the reaction, the obtained TGA coated Pt: CdTe QDs precipitate was washed with ethanol several times to remove the excess contaminants.

### Preparation of the Ab_2_-Labeled Probe (Ab_2_-Pt: CdTe QDs)

To the Pt: CdTe QDs conjugated with anti-HER2, 1.0 ml of Pt: CdTe QDs was activated by adding EDC (500 μl of 5 mg ml^−1^) and NHS (500 μl of 0.25 mg ml^−1^). The mixture was rotated for 15 min at room temperature and then added with 500 μl of anti-HER2 (50 μg ml^−1^) and oscillated at 4°C overnight. Finally, it was stored at 4°C.

### Construction of the Immunosensor

**SCHEME 1:**
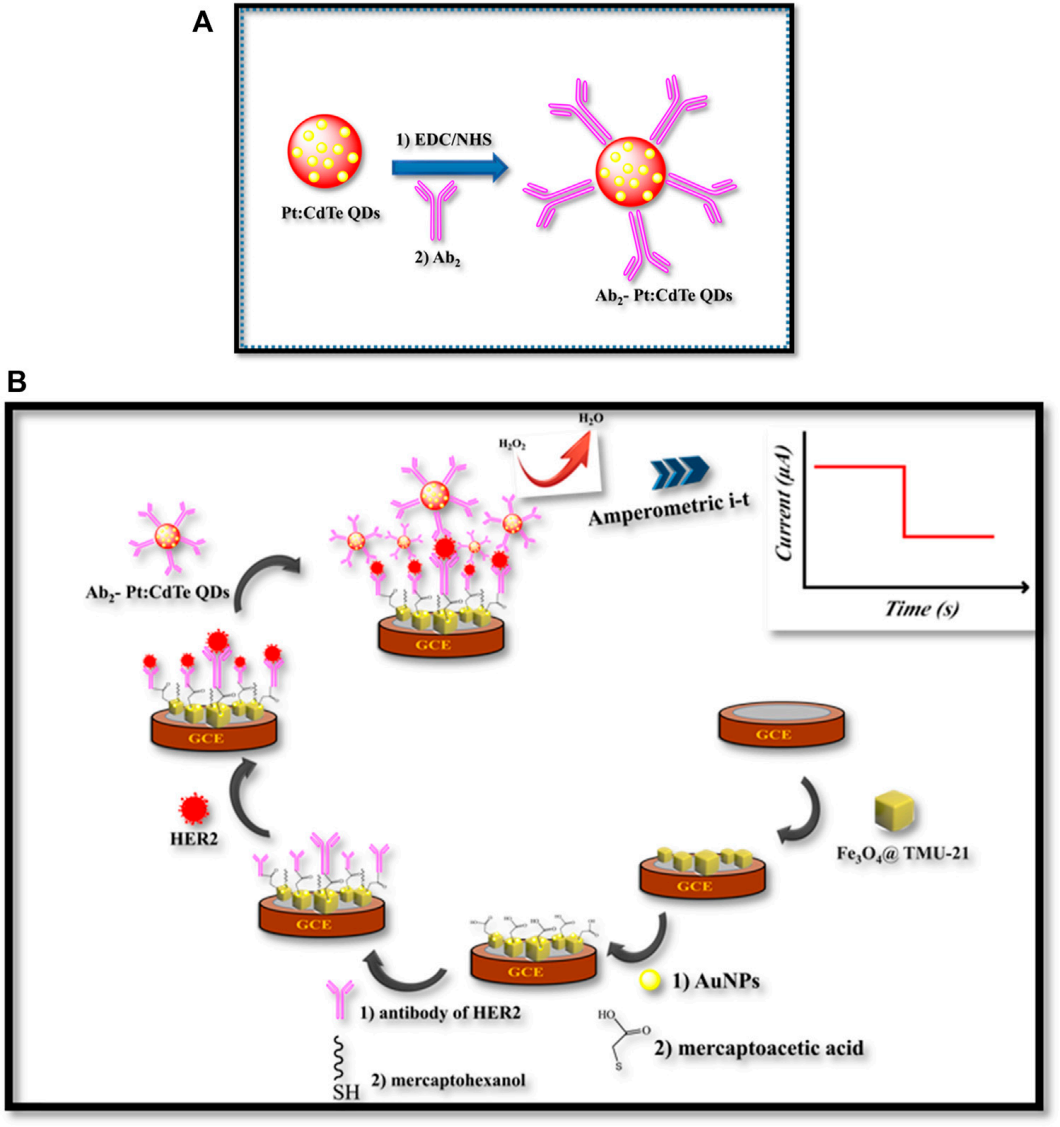
**(A)** The illustration of the preparation process of Ab_2_- Pt:CdTe QDs, **(B)** fabrication of the sandwich-type electrochemical immunosensor

Before modification, the GCE was polished successively with (1.0 and 0.05 μm) Al_2_O_3_ water slurry using a polishing cloth, and it was rinsed with doubly distilled water. Then, 1 mg Fe_3_O_4_@TMU-24 was dispersed in 10 ml DMSO containing 2.5% (V/V) Nafion with ultra-sonication for 1 h. Then, 4 µl of the suspension was dropped on the surface of the GCE and dried at room temperature to obtain GCE/Fe_3_O_4_@. Then, the AuNPs were electrodeposited onto the GCE/Fe_3_O_4_@ by CV scanning from −0.2–1.2 V in a solution of 0.5 M H_2_SO_4_ containing 0.6 M of HAuCl_4_ at a scan rate of 50 mV s^−1^ for 15 cycles. The constructed electrode was immersed in a 40 mM MAA solution for 5 h to form a self-assembled monolayer*.* Then, the prepared electrode (GCE/Fe_3_O_4_@-AuNPs/MAA) was rinsed carefully with double distilled water to remove physically adsorbed MAA and MAA carboxyl groups were activated on the electrode surface using EDC/NHS. Then, the electrode was then washed with PBS and immersed in a solution of Ab_1_ (4 μg ml^−1^) for 40 min, then, was washed with PBS. Then, to block the residual active sites on the working electrode it was immersed in the 3 ml mercaptohexanol for 30 min, followed this step, the GCE/Fe_3_O_4_@-AuNPs/MAA was washed with PBS. The obtained immunosensor was stored at 4°C for later use (Scheme 1).

### Sensing Procedure

The performance of the immunosensor for sensing the HER2 was carried out by the Amperometric technique with 5.0 μl of HER2 varying concentrations after 45 min incubation at 37°C. By using 0.8 mg ml^−1^ Ab_2_-Pt: CdTe QDs (Ab_2_ labeled probe) attached to the surface of GCE/Fe_3_O_4_@-AuNPs/MAA/Ab_1_/HER2 by the specific binding of antigen and antibody, thereby catalyzing H_2_O_2_ to obtain the higher reducing current signals. For the determination of HER2, a constant voltage of -0.4 V, the H_2_O_2_ (10 μl, 5.0 mM) was stirred with a constant voltage of −0.4 V, and the amperometric signal was then collected. The CV of the biosensor was scanned in 0.1 M KCl containing 5.0 mM of [Fe(CN)_6_]^3−/4−^, which was used as the electrochemical probe. The Nyquist plots were taken under the frequency ranging from 10^5^ to 0.1 Hz and AC amplitude 5 mV in the solution of 0.1 M KCl containing 5.0 mM of [Fe(CN)_6_]^3−/4−^ as the probe and then the charge transfer resistance (Rct) was tracked.

## Results and Discussion

### Characterization of Fe_3_O_4_@ TMU-24

**FIGURE 1:**
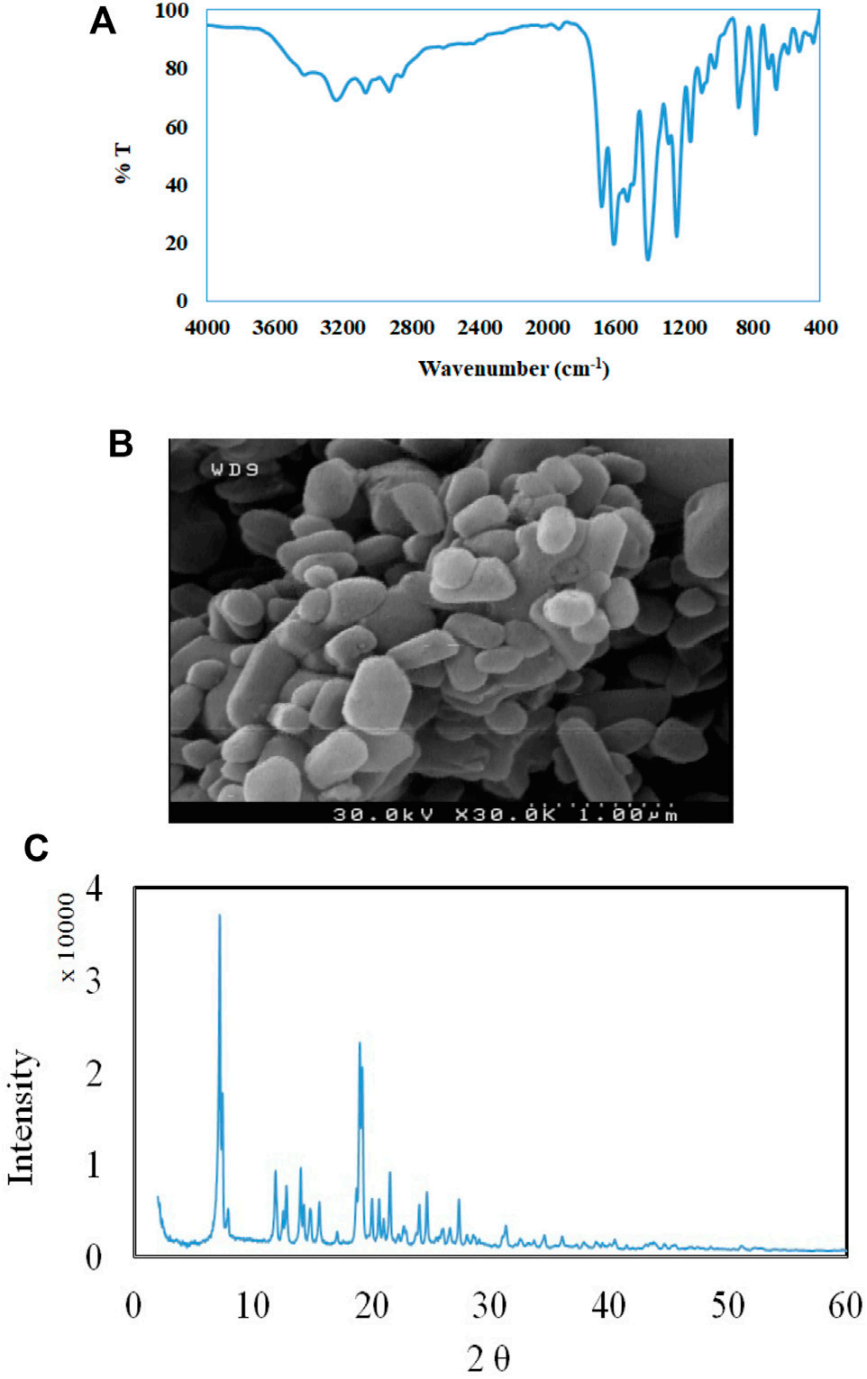
**(A)** FT-IR spectra, and **(B)** SEM image of TMU-24 **(C).** XRD patterns of TMU-24.

The FT-IR spectroscopy of TMU-24 is presented in Figure 1A. The peaks at 1,378 and 1,427 cm^−1^ referring, as C=C bonds, appear in TMU-24. Peaks at 2,752 and 2,851 cm^−1^ corresponded to the stretching vibration of C–H bonds in the benzene ring. The peak at 1,611 cm^−1^ is attributed to the stretching vibration adsorption peaks of the C=O bonds.

The morphology of TMU-24 was investigated by SEM. As shown in Figure 1B, the particle of nanomaterials is presented XRD patterns are similar to the previously reported data that revealed syntheses of TMU-24 (Figure 1C). Based on the SEM images, the average diameter of the TMU-24 studied here is about 350 nm.

From N_2_ isotherm at 77 K, TMU-24 was found to be nonporous materials with negligible uptake, which reveal an “N_2_-phobic” behavior. We reasoned that this behavior could be due to the existence of different structural rearrangements depending on the MOF during sorption or when exposed to cryogenic temperatures and/or under vacuum, reducing/preventing access to the porosity. However, other factors cannot be fully excluded, such as the existence of strong interactions between N_2_ and the channel walls at 77 K that hinder diffusion into the material. Interestingly, contrariwise to the adsorption of N_2_, the CO_2_ sorption isotherm revealed that TMU-24 is porous, and CO_2_ uptake at 203 K was calculated to be 6.3 mmol g^−1^ with the pore volume of 0.22 cm^3^ g^−1^ at 760 Torr.

### Characterization of Synthesized TGA-Capped Pt: CdTe QDs

**FIGURE 2:**
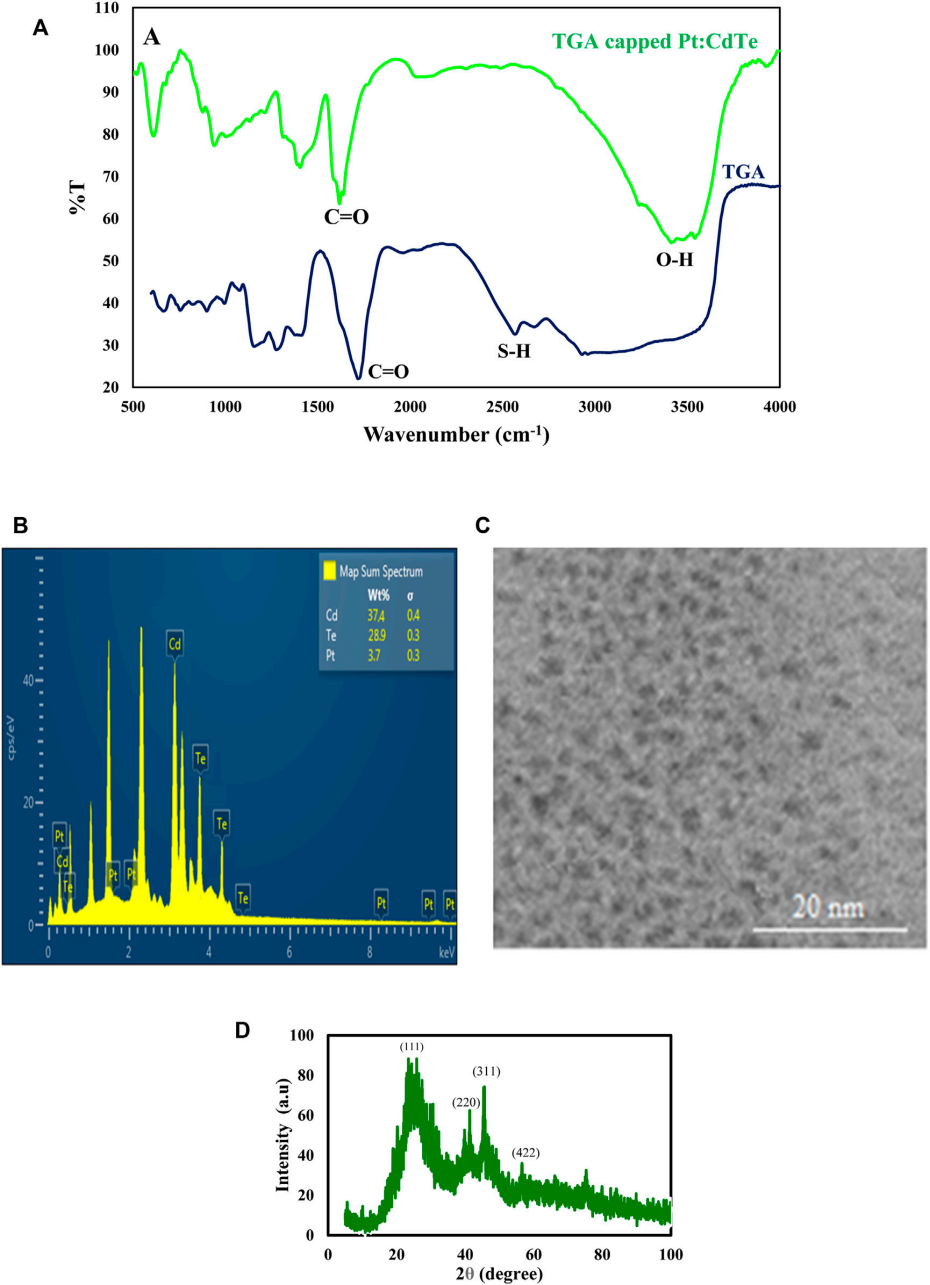
**(A)** FT-IR spectra of Pt: CdTe NPs and TGA **(B,C)** TEM image and EDX spectrum of Pd: CdTe NPs **(D)** XRD patterns of Pt:CdTe QDs.

FT-IR spectroscopy was applied to explore the surface functionalization of the TGA capped Pt: CdTe and TGA. Figure 2A shows the FT-IR spectra of TGA-capped Pt: CdTe QDs and TGA alone. In TGA-capped Pt: CdTe QDs spectrum a broad and strong peak at 3,441 cm^−1^ was observed assigning to the stretching vibrations of O-H while this peak is not observed in TGA. Instead, the stretching vibration of the S-H bond and C=O in TGA appeared at 2,565 cm^−1^ and 1716 cm^−1^, which in the spectrum of TGA-capped Pt: CdTe QDs the first peak was disappeared that may be due to the binding of metal cations with thiol group and the second peak was shifted to 1,578 cm^−1^. These results confirmed the attachment of TGA onto the quantum dots.

The particle size and morphology of the as-prepared TGA-capped Pt: CdTe QDs were studied by the TEM method. As shown in Figure 2B the prepared QDs with nearly spherical morphology have a uniform size with a diameter of about 2 nm. The elemental composition of the TGA capped Pt: CdTe d-dots NPs was corroborated by EDX analysis (Figure 2C). The EDX indicates the presence of Pt, Cd, and Te in the prepared QDs.

The crystal structure of undoped Pt: CdTe QDs were investigated with an XRD pattern and is shown in (Figure 2D). The XRD pattern achieved for the Pt: CdTe QDs illustrates four peaks positioned at 2θ = 22.57°, 39.70°, 45.82°, and 57.54° that are corresponding to (111) (220), (311), and (422) planes, which their positions are consistent with the values of the standard diffraction patterns of the cubic structure of CdTe (JCPDS no. 03-065-1,046).

### Characterization of Pt: CdTe QDs and Labeled Ab_2_-Pt: CdTe QDs

**FIGURE 3:**
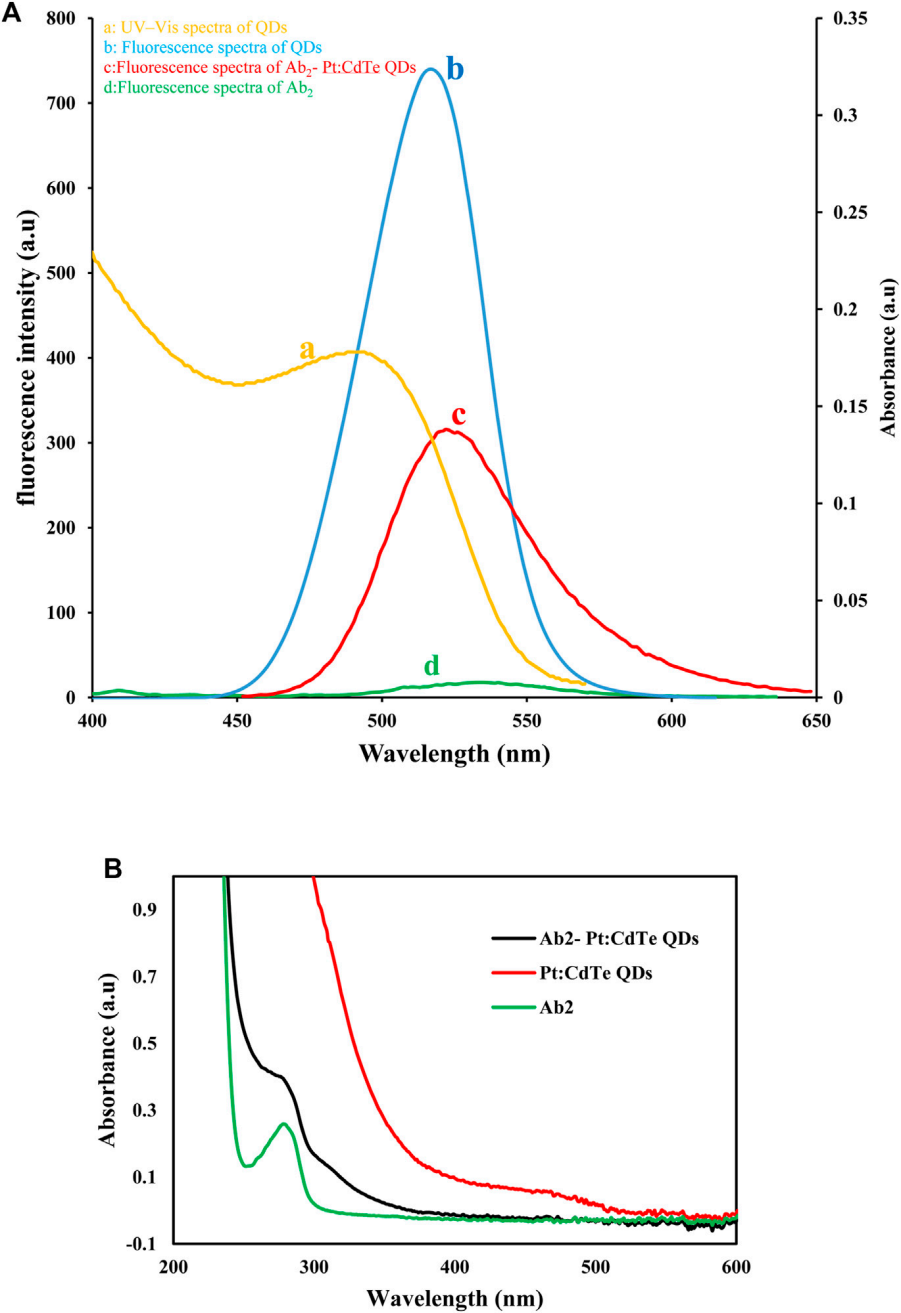
UV–Vis spectra of QDs and Fluorescence spectra of QDs and Ab_2_- Pt:CdTe QDs.

The UV–Vis absorption and fluorescence spectra of Pt: CdTe QDs were presented in Figure 3A. As seen in Figure 3A curve the absorption spectrum of Pt: CdTe QDs was observed with the excitonic at 495 nm and it is a wide spectrum The Pt: CdTe QDs fluorescence was emitted at 515 nm upon excitation at 360 nm (curve b). The emission of Pt: CdTe QDs was close to its absorption onset indicating the emission arising from the direct recombination of charge carriers between conduction and valence bands.

To confirm the binding of Pt: CdTe QDs to Ab_2_, the fluorescence spectra of Pt: CdTe QDs, Ab_2_, and Ab_2_- Pt: CdTe QDs were also recorded and compared with the emission spectra of Pt: CdTe QDs. As Figure 3A shows, after, the attachment of Ab2 to QDs and covering the surface of QDs with Ab_2_, the related fluorescent signal decreases and shows a redshift at 525 nm which can prove the interaction between Ab_2_ and QDs due to their conjugation.

Similar to UV-Vis studies, fluorescence studies show the same trend. The QDs have an intense band of fluorescence at 515 nm, while the Ab_2_ has almost no band at all (Figure 3B). After the attachment of Ab_2_ to QDs, the related fluorescent signal decreases with a redshift to 525 nm. These studies suggest that the Ab_2_-QDs are successfully formed.

### The Morphology and Electrochemical Properties of the Electrode

**FIGURE 4:**
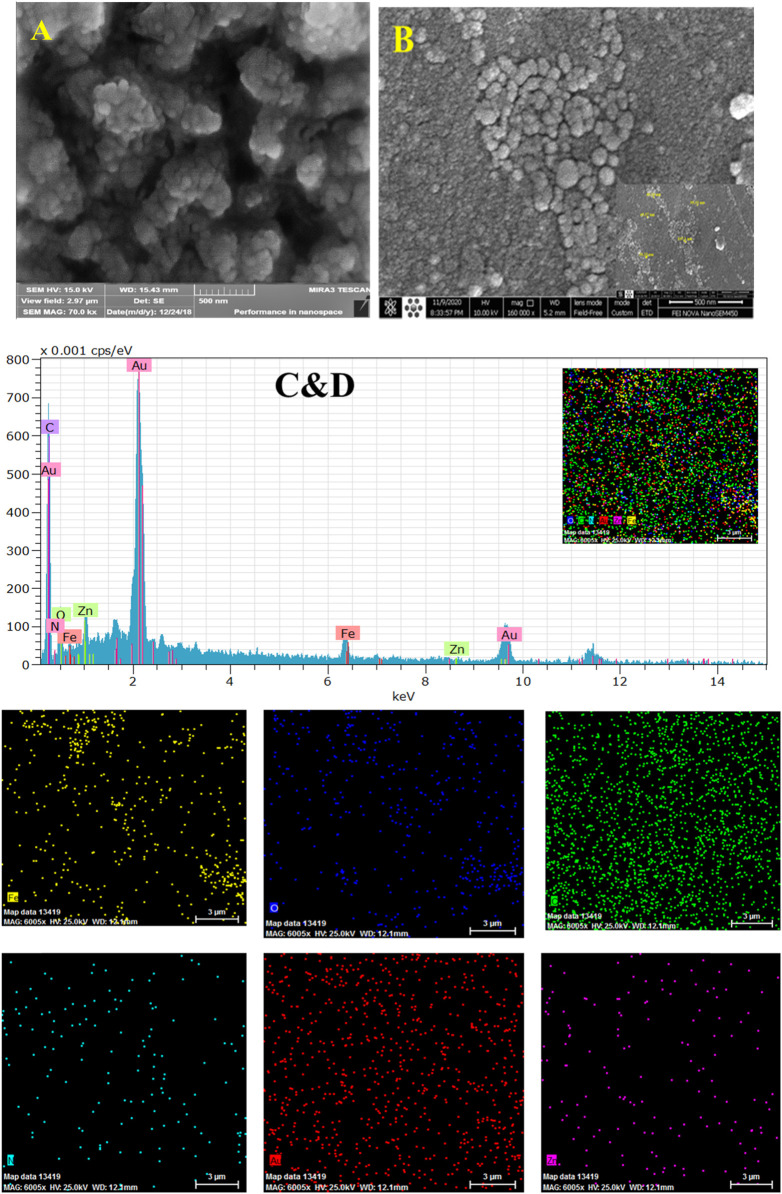
SEM images of GCE/Fe_3_O_4_@ TMU-24 **(A)**, and GCE/Fe_3_O_4_@ TMU-24 -AuNPs **(B)**, EDX mapping and EDX spectrum recorded at GCE/Fe_3_O_4_@ TMU-24 -AuNPs elemental different showing signal of Au, C, O, N, Fe, and Zn respectively, as is illustrated **(C,D)**.

The surface morphologies of the constructed immunosensor during modifications were characterized by the SEM as an excellent procedure. Figure 4 shows that the composite film of Fe_3_O_4_@ and Nafion is covered the surface of GCE. The size of Fe_3_O_4_@ with a diameter of about 50–100 nm with the ball shape in the Nafion film is distinguishable in this image. The distribution of the Au NPs with an average diameter of about 36 nm at the Fe_3_O_4_@ surface is illustrated in Figure 4B. This film by its nanostructure morphology can increase the surface area of the electrode, which in turn can increase a biosensor, especially for immobilization of an antibody.

**FIGURE 5:**
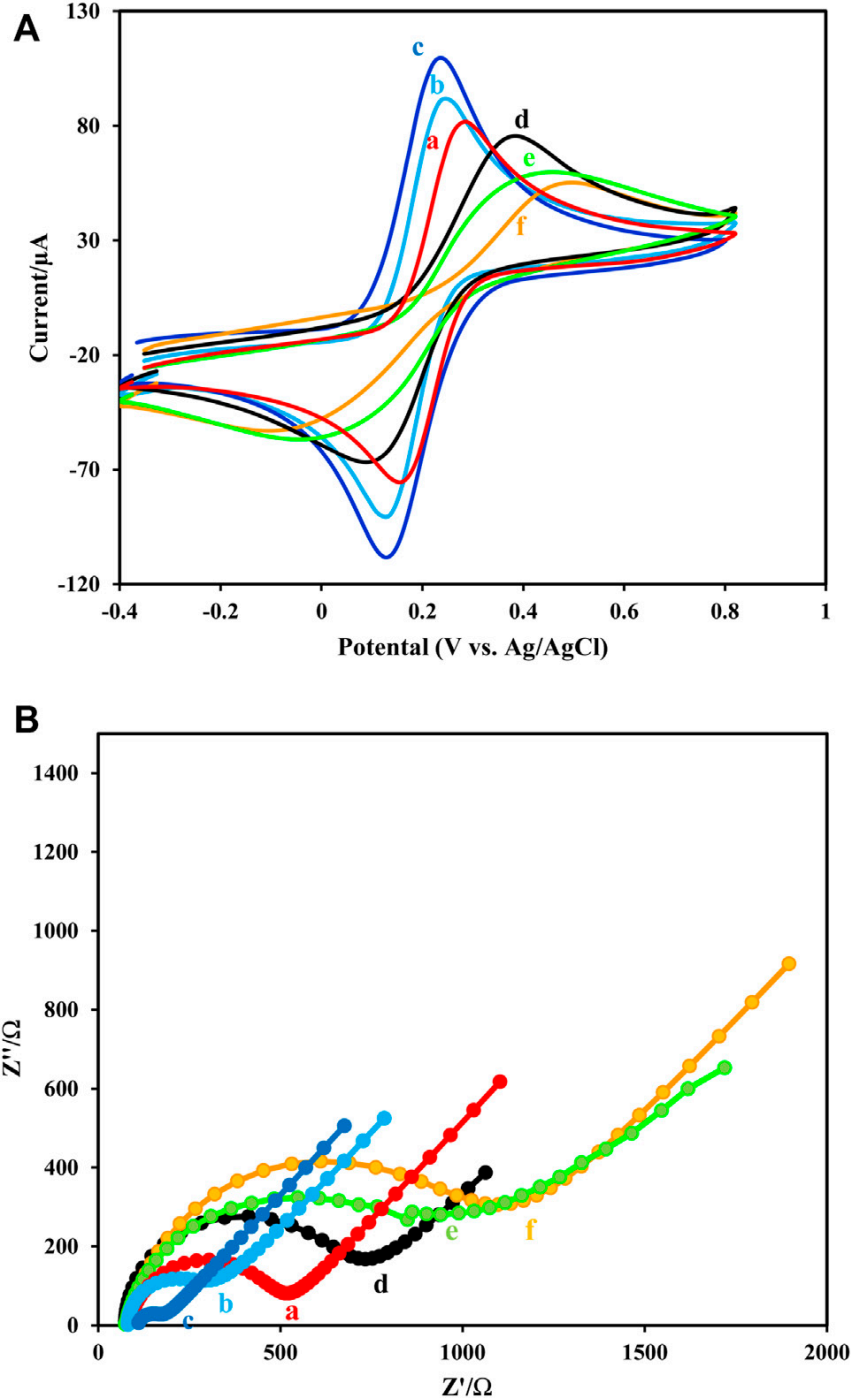
**(A,B)** Cyclic voltammograms and **(B)** Nyquist diagrams of 5.0 mM [Fe(CN)_6_]^3-/4-^ (0.1 M KCl) and scan rate of 50 mV s^−1^recorded at (a) GCE, (b) GCE/Fe_3_O_4_@ TMU-24, (c) GCE/Fe_3_O_4_@ TMU-24-AuNPs, (d) GCE/Fe_3_O_4_@ TMU-24 -AuNPs/MCA, (e) GCE/Fe_3_O_4_@ TMU-24 -AuNPs/MCA/Ab_1_, (f) GCE/Fe_3_O_4_@ TMU-24 -AuNPs/MCA/Ab_1_/HER2.

The EDX images (Figure 1B) and elemental mapping (Figure 1C) indicate the presence and dispersion of different elements of Au, C, O, N, Fe, and Zn at the prepared composite of elements at the platform immunosensor (Figure 4C). The CV and EIS techniques were used to monitor the surface changes during the construction process of the HER2 sandwich assay. Figure 5 shows different CVs of bare GC (curve a), GC/Fe_3_O_4_@ TMU-24 (curve b), GC/Fe_3_O_4_@ TMU-24 –AuNPs/MAA (curve c), GC/Fe_3_O_4_@ TMU-24 -Au NPs/MAA/Ab_1_ (curve e), GC/Fe_3_O_4_@ TMU-24-AuNPs/Ab_1_/HER2 (curve f) electrodes. In the solution of 5.0 mM [Fe(CN)_6_]^3-/4-^ (0.1 M KCl) and a scan rate of 50 mV s^−1^, the bare GC electrode shows well-known reversible peaks of redox probes. After the position of Fe_3_O_4_@ TMU-24 on the GC electrode surface, the peak current is increased and the peak-to-peak separation (ΔE_p_) is decreased, which can be contributed to the electron transfer process being facilitated by using Fe_3_O_4_@ TMU-24 as an excellent signal amplifying material.

Electrodeposition of AuNPs on GCE/Fe_3_O_4_@ TMU-24 increased the value of the redox current of the probe, which clearly shows the deposition of Au NPs in the resulted electrode. This nanoparticle improves the electrode conductivity and facilitates the kinetic electron transfer. Next, by appending the MAA on GCE/Fe_3_O_4_@ TMU-24-AuNPs, the semicircle diameter is also dramatically, decreased from the current value, indicating MAA as a low conductive film.

As was expected, after immobilization of Ab_1_ in GCE/Fe_3_O_4_@ TMU-24-AuNPs/MAA/Ab_1_, the current is dramatically decreased and the current decrease is continued by the addition of antigen. These observations prove that the surface GC/Fe_3_O_4_@ TMU-24-AuNPs/MAA was successfully modified with Ab0_1_. The CV was used to monitor the surface changes during the construction process. The results of the CV are in good agreement with the results of the EIS.

TMU-24 exhibited excellent electrochemical behavior to facilitate the electron transfer and prominent redox properties by charge compensation. Furthermore, it existed a large surface area and high affinity to AuNPs in terms of strong interaction between -CN and AuNPs.

### Study of the Electrocatalytic Activity of the Working Electrode Fabrication Toward H_2_O_2_

**FIGURE 6:**
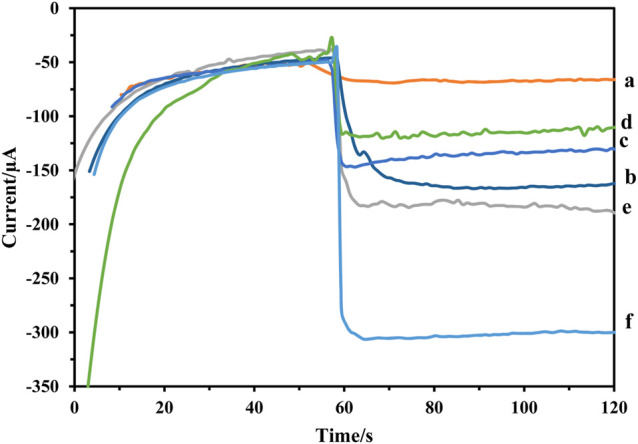
Amperometric response of the **(A)** GCE, **(B)** GCE/Fe_3_O_4_@ TMU-24 -AuNPs,**(C)** GCE/Fe_3_O_4_@ TMU-24 -AuNPs/MCA/Ab_1_, **(D)** GCE/Fe_3_O_4_@ TMU-24 -AuNPs/MCA/Ab_1_/HER2, **(E)** GCE/Fe_3_O_4_@ TMU-24 -AuNPs/MCA/Ab_1_/HER2/CdTe QDs -Ab_2_, and **(F)** GCE/Fe_3_O_4_@ TMU-24 -AuNPs/MCA/Ab_1_/HER2/Pt:CdTe QDs -Ab_2_.

The amperometric was used to record the steps of immunosensor fabrication. As Figure 6 shows, bare GCE did not show any catalytic effect on the reduction of H_2_O_2_ (curve a) and GCE/Fe_3_O_4_@ TMU-24-AuNPs had a large current signal (curve b). While, the current signal declined after Ab_1_, and HER2 were modified on the surface (curves c and d), successively. It can be attributed to proteins that block the electroactive material from coming into contact with the platform. As result, confirms the formation of the antigen-antibody complex. As a result, to produce high-sensitivity signals, the catalytic activity of Pt: CdTe QDs and CdTe QDs in the label of Ab_2_ were compared. As demonstrated in Figure 6, the current response of Pt: CdTe QDs -Ab_20_ (curve f) was greater than CdTe QDs-Ab_2_ (curve e). The experiment result confirmed that.

Pt: CdTe QDs-Ab_2_ possessed efficient catalytic activity towards H_2_O_2_. Doping Pt to CdTe QDs has led to a significant increase in the immunosensor response signal. As a result, Pt can act as catalytic labels for the reduction of H_2_O_2_ to amplify the signal of the electrochemical immunosensor. Therefore, when the HER2 is sandwiched by the Ab_1_ and Pt: CdTe QDs -Ab_2_, the current signal increased rapidly. It can be attributed to Pt: CdTe QDs -Ab_2_ with high catalytic activity. Therefore, the immunosensor was fabricated successfully.

### Optimization of Experimental Condition

We optimized the following parameters for maximum performance of the immunosensor: incubation times of Fe_3_O_4_@ TMU-24-AuNPs/Ab_1_ with HER2 and Ab_2_- Pt: CdTe QDs/HER2 with pH. The incubation time effect of 10 ng ml^−1^ of HER2 on the surface of GC/Fe_3_O_4_@ TMU-24-AuNPs/MAA/Ab_1_ electrode on signal response was evaluated. As it can be seen in Supplementary Figure S1A, the immunosensor response increases with time raise and after 40 min the signal is almost unchanged, the maximum signal response was obtained then it became almost saturated. Therefore, the time of 40 min was chosen as the incubation time for determining the HER2.

The incubation time of Ab_2_- Pt: CdTe QDs labeled on the surface of GCE/Fe_3_O_4_@ TMU-24-AuNPs/MCA/Ab_1_/HER2 was evaluated with the same amperometric method (Supplementary Figure S1B) proves, the incubation time of Ab_2_- Pt: CdTe QDs labeled 40 min was selected and applied for HER2 biomarker analysis.

The pH of PBS buffer has a high influence on the electrochemical behavior of the stabilized peptide. The pH optimization of PBS solution was evaluated from pH 5.5 to 9.5. The amperometric response of the immunosensor increased up to 7.5 and then decreased. The maximum signal response of the sensor was obtained at pH 7.5 in Supplementary Figure S1C, which is close to the isoelectric point of the protein. Therefore, pH 7.5 was selected for the following.

### Analytical Performance

**FIGURE 7:**
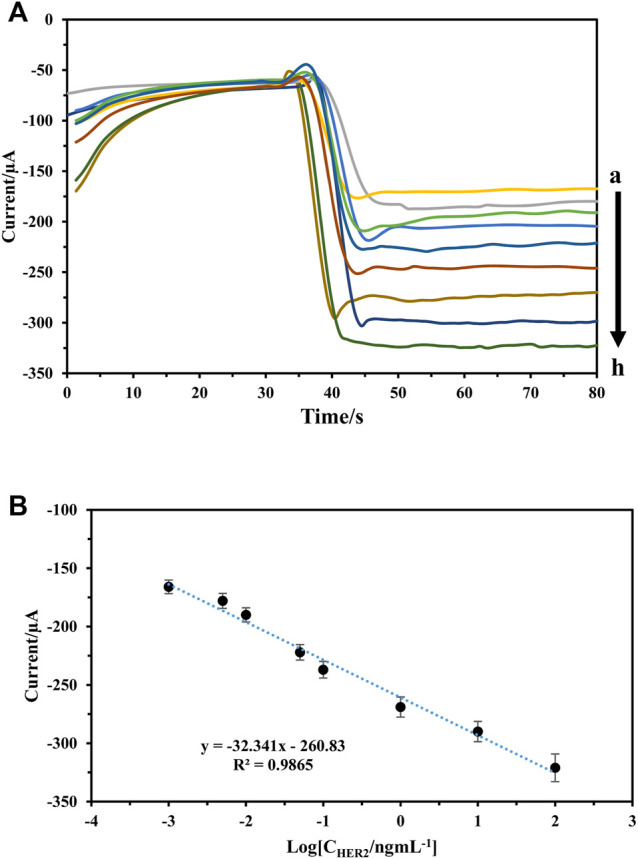
**(A,B)** Amperometric response of the immunosensor to different concentrations of HER2, from a to )1 pg/ml, 5 pg/ml, 10 pg/ml, 50 pg/ml, 100 pg/ml, 1 ng/ml, 10 ng/ml, and 100 ng/ml **(B)** calibration curve of the immunosensor to different concentrations of HER2.

In optimum conditions, the analytical performances of immunosensors were evaluated for different concentration detection of HER2 using Pt: CdTe QDs as labels. The amperometric’s signals of the immunosensor were recorded at −0.4 V in pH 7.0 PBS. As Figures 7A,B show, the increase in incubation of HER2 at the sensor surface caused a gradual increase in amperometric signals (Figure 7A). The amperometric current was charted against of logarithm of HER2 concentration and the calibration chart had shown a linear relationship in the range of 1 pg ml^−1^–100 ng ml^−1^ with a regression equation of I = -32.34 log (C ng·mL^−1^) + 260.8 (*R* ^2^ = 0.9865) (Figure 7B) and an appropriate response with a detection limit of 0.175 pg ml^−1^. The proposed immunosensor exhibits a comparable and even better performance for the determination of HER2 relative to that of the reported sensors.

A comparison between immunosensor presented in previous studies has been shown in Table 1. The proposed immunosensor has a decent function (For example, lower detection limit, and wide linear range).

**TABLE 1:** Comparison of different methods for HER2 detection.

Label	Method	Liner range	Limit of detection	References
Label free	CV&DPV	10–70 ng ml^−1^	1.6 ng ml^−1^	Pacheco et al. (2018)
Label free	DPV	10–5 × 10^6^ cells mL^−1^	2 cells mL^−1^	Salahandish et al. (2018)
Ab2-PbS QDs^a^	SWV	1.0–100 ng ml^−1^	0.28 ng ml^−1^	Ahmad et al. (2019)
CdSe@ZnS	DPV	10–150 ng ml-1	2.10 ng ml-1	(Freitas et al., 2020)
Label free	DPV	10 pg ml^−1^–10 ng ml^−1^	0.99 pg ml^−1^	Emami et al. (2014)
		10–100 ng ml^−1^		
Label free	EIS	10.0 pg ml^−1^–100 ng ml^−1^	10.00 pg ml^−1^	Sharma et al. (2018)
Hyd−AuNP−Apt^c^	SWSV^d^	0.1 pg ml^−1^–10 ng ml^−1^	37.00 pg ml^−1^	Zhu et al. (2012)
Label free	Amperometric	1.0 pg ml^−1^–100 ng/ml^−1^	0.30 pg ml^−1^	(Ehzari et al., 2020d)
Pt: CdTe QDs	Amperometric	1.0 pg mL^−1^–100 ng ml^−1^	0.17 pg ml^−1^	Present work

### Evaluation of Stability, Reproducibility, and Specificity

**FIGURE 8:**
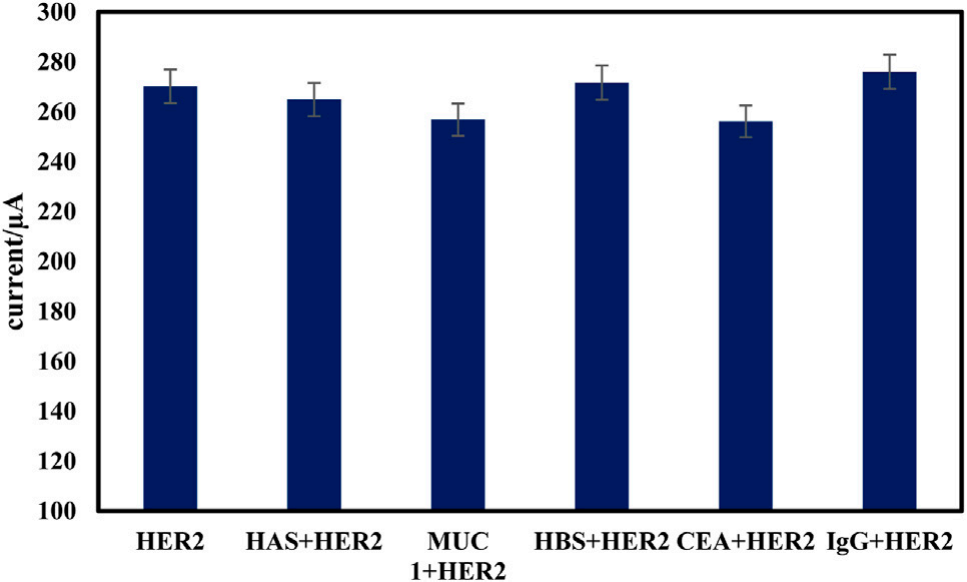
Specificity of the proposed electrochemical immunosensor. The amperometric signal changes of the immunosensor to 10 ng/ml HER2 and in presence of 100 ng/ml CEA, HSA, HBS, and IgG.

The stability of the proposed immunosensor was evaluated by monitoring of signal of 10 ng ml^−1^ of HER2 stored at 4°C in 0.1 M PBS of pH 7.0 solution. After one week no change, but 2 weeks of storage under similar conditions reduced the initial current of immunosensor by about 12%. The stability of the sensor is attributed to the presence of Fe_3_O_4_@ TMU-24 as a robust sublayer, which can assist the composite to keep intact the established architecture in stabilizing the probe connected to the electrode surface. The relative standard deviations (RSD) of six immunosensor were found to be 3.15% suggesting the acceptable reproducibility of the electrode. The binding specificity of the proposed immunosensor to 10 ng ml^−1^ of HER2 was compared with those obtained by equal concentration (100 ng ml^−1^) of HSA, hepatitis B surface antigen (HBS), carcinoembryonic antigen (CEA), and human immunoglobulin (IgG) and the results are presented in Figure 8. As shown in Figure 8, in the presence of interfering species, the current response to HER2 did not change. These results indicate that the constructed sensor is suitable for sensing HER2.

The main advantage of using the Fe_3_O_4_@ TMU-24-AuNPs/Ab1 is easy and quick surface renewal after each use. Thus, the repeatability of the response signal was studied for HER2 biomarker by an amperometric method in optimized conditions. The relative standard deviation (RSD) of 1.65% was obtained.

### Clinical Application of the Proposed Immunosensor

To show the use of constructed immunosensors in the analysis of real samples, accurate HER2 determination in human serum samples was measured by the standard addition method following the addition of different concentrations of HER2 into healthy human serum samples. The results obtained from human serum samples are assembled in Table 2. The accuracy and precision of the proposed immunosensor were evaluated by recovery and the relative standard deviation calculation respectively. The recovery of the amounts of HER2 detected by the proposed immunosensors was in the range of 98 to 103.7% and the relative standard deviations were obtained to less than 5% that revealing the utilization potentiality of the assay of HER2 based on the designed sensor using Pt: CdTe QDs labeled Ab_2_ for clinical analysis samples.

**TABLE 2:** Determination of added HER2 to human blood serum.

Number of sample	Added (ng/ml^−1^)	Found (ng/ml^−1^)	RSD (%)	Recovery (%)
1	—	0.03	1.25	—
2	1	1.10	3.77	107.0
3	5	4.93	3.60	98.0
4	10	10.40	4.35	103.7

## Conclusion

This study presented a highly accurate and precise sandwich-type electrochemical immunosensor for HER2 detection based on a novel Pt: CdTe QDs-labeled antibody as a bio probe. In sensor architecture, the unique properties of Fe_3_O_4_@ TMU-24 were used to stabilize the antibody and amplify the signal. The QDs labeled antibody probe could well track the biomarkers of HER2. The results show proposed immunosensor has good performance for HER2 detection with a wide linear range of 1 pg ml^−1^–100 ng ml^−1^and a low limit of detection of 0.175 pg ml^−1^. Most importantly, the simple performance and sensitivity of this sensor had shown promising prospects in clinical trials.

## Data Availability

The original contributions presented in the study are included in the article/Supplementary Material, further inquiries can be directed to the corresponding author.
