# Supplementary material for: A Sandwich-Type Electrochemical Immunosensor Using Antibody-Conjugated Pt-Doped CdTe QDs as Enzyme-Free Labels for Sensitive HER2 Detection Based on a Magnetic Framework
Source: Front Chem. 2022 Jun 9;10:881960. doi: 10.3389/fchem.2022.881960 (PMC9218600; doi:10.3389/fchem.2022.881960)
Supplement: Supplementary file 1 [file DataSheet1.docx]

**Fig.** S**1.** Effects of (A) the incubation time of HER2, (B) the incubation time of Ab_2_ labeled probe, and pH of solution on the immunosensor response to 1 ng mL^-1^ HER2.
